# Osteoprotegerin (OPG) Upregulation Activates Breast Stromal Fibroblasts and Enhances Their Pro-Carcinogenic Effects through the STAT3/IL-6 Signaling

**DOI:** 10.3390/cells11213369

**Published:** 2022-10-25

**Authors:** Huda K. Al-Nasrallah, Mysoon M. Al-Ansari, Abdelilah Aboussekhra

**Affiliations:** 1 Department of Molecular Oncology, King Faisal Specialist Hospital and Research Center, Riyadh 11211, Saudi Arabia; 2 Department of Microbiology, Faculty of Science and Medical Studies, King Saud University, Riyadh 11451, Saudi Arabia

**Keywords:** breast cancer, cancer-associated fibroblasts, OPG, cancer stem cells

## Abstract

Breast carcinomas are composed of cancer cells surrounded by various types of non-cancer cells such as fibroblasts. While active cancer-associated fibroblasts (CAFs) support tumor initiation and progression, quiescent breast stromal fibroblasts (BSFs) inhibit these effects through various cytokines such as osteoprotegerin (OPG). We showed here that OPG is upregulated in CAFs as compared to their adjacent normal tumor counterpart fibroblasts. Interestingly, breast cancer cells can upregulate OPG in BSFs in an IL-6-dependent manner through the IL-6/STAT3 pathway. When upregulated by ectopic expression, OPG activated BSFs through the NF-κB/STAT3/AUF1 signaling pathway and promoted their paracrine pro-carcinogenic effects in an IL-6-dependent manner. In addition, this increase in the OPG level enhanced the potential of BSFs to promote the growth of humanized orthotopic tumors in mice. However, specific OPG knock-down suppressed active CAFs and their paracrine pro-carcinogenic effects. Similar effects were observed when CAF cells were exposed to the pure recombinant OPG (rOPG) protein. Together, these findings show the importance of OPG in the activation of stromal fibroblasts and the possible use of rOPG or inhibitors of the endogenous protein to target CAFs as precision cancer therapeutics.

## 1. Introduction

Breast carcinoma is the most common cancer in women worldwide [1]. Cancer cells are present in the tumor microenvironment (TME), which is composed of acellular as well as different cellular components such as extracellular matrix (ECM) and various types of cells, including fibroblasts, immune cells, inflammatory cells, and endothelial cells. Cancer-associated fibroblasts (CAFs) are the most active type of cells in the stromal compartment [2]. During breast tumorigenesis, normal fibroblasts are affected by many factors from cancer cells, which generate active pro-tumorigenic fibroblasts [3,4,5].

In contrast to quiescent fibroblasts, which have potent anti-neoplastic activities, activated CAFs exhibit enhanced proliferative and migratory properties [6,7,8,9]. Breast CAFs are heterogeneous and divided into subsets characterized with distinct properties and levels of activation, with α-SMA and FAP-α being the main markers [10,11,12]. CAFs play critical roles in tumor initiation, angiogenesis, dissemination, and metastasis through the production of multiple ECM proteins and regulatory molecules [3,4,5,13].

Osteoprotegerin (OPG), also known as FDCR-1, TR1, and OCIF, is a decoy receptor for Receptor Activator of NF-κB Ligand (RANKL) and a member of the TNF receptor superfamily [14,15]. The OPG protein is encoded by the *TNFRSF11b* gene and has both intra and extracellular functions [14]. Besides being a key regulator of bone metastasis, OPG is also associated with breast tumorigenesis [16]. Indeed, OPG has been found to be highly expressed in more than 50% of primary breast cancer cells, and in invasive and inflammatory breast cancer tissues [17,18]. Interestingly, while OPG has autocrine oncogenic functions, it has tumor suppressive functions when secreted or used as recombinant pure protein [19]. Furthermore, we have recently shown that the secreted OPG from normal breast stromal fibroblasts as well as the recombinant protein inhibit breast carcinogenesis both in vitro and in vivo [6]. Strikingly, OPG expression seems to be associated with both good and poor patient survival [20]. In the present report, we investigate the role of OPG in the activation and the treatment of breast stromal fibroblasts (BSFs). We show that OPG is upregulated in CAFs relative to their adjacent normal tumor counterpart fibroblasts (TCFs) isolated from the same patients, and ectopic expression of OPG activates BSFs. Interestingly, recombinant human OPG (rOPG) normalized the active features of breast CAFs.

## 2. Materials Methods

### 2.1. Cells, Cell Culture and Reagents

Breast fibroblast cells were obtained and used as previously described [21]. NBFs (normal breast fibroblasts) are cultures obtained from tissues derived from reduction mammoplasty, CAFs (cancer-associated fibroblasts), and TCFs (tumor counterpart fibroblasts) are from histologically normal parts of tumors. Fibroblasts were cultured in M199 medium (Gibco, Grand Island, NY, USA ) and Ham’s F12 (Gibco) mixed 1:1 and supplemented with 20% Heat Inactivated Fetal Bovine Serum (FBS) (Gibco) and 1% Antibiotic-Antimycotic 100X (Gibco). Breast cancer cells MDA-MB-231 and MCF-7 were obtained from ATCC and were cultured in RPMI 1640 medium (Gibco) supplemented with 10% FBS and 1% Antibiotic-Antimycotic, while Human Mammary Luminal Epithelial (HMLE) cells were a generous gift from Dr. Hazem Ghebeh (KFSHRC) and were maintained in DMEM/F12 (Gibco) supplemented with 0.5% Bovine Pituitary Extract (Gibco) and 1% HuMEC supplement (Gibco) in addition to 2% FBS and 1% Antibiotic-Antimycotic. Cells were maintained at 37°C in humidified incubator with 5% CO_2_. For long storage, cells were kept in freezing media (Gibco). Recombinant IL-6 was purchased from GenWay Biotech. Recombinant OPG was purchased from Sigma-Aldrich.

### 2.2. RNA Purification and qRT-PCR

Total RNA was purified using RNeasy Mini Kit (Qiagen, Manchester, UK) according to the manufacturer’s instructions. RNA was quantified using Thermo Scientific NanoDrop ND-1000 Spectrophotometer and 1 μg was used for reverse-transcription using random primers (Clontech Laboratories-Advantage RT-for-PCR Kit) (Clontech Laboratories, Mountain View, CA, USA) to make the cDNA following the manufacturer protocol. For qRT-PCR, the FastStart essential DNA Green Master 2X (Roche, New York, NY, USA) and specific primers were used and the amplifications were performed on the LightCycler 96 Real-Time PCR System (Roche). Primer sequences are as shown in Supplementary Table S1.

### 2.3. siRNA Transfection

STAT3 siRNA was obtained from Qiagen, OPG siRNA and control siRNA were obtained from (Santa Cruz, Santa Cruz, CA, USA). The transfections were carried out using the Lipofectamine RNAiMAX transfection reagent (Invitrogen, Carlsbad, CA, USA) following the protocol recommended by the manufacturer.

### 2.4. Transfections with Plasmids

Plasmid transfection was carried out using OPG-ORF and the corresponding control (Origene, Rockville, MD, USA) at a final concentration of 3 µg using Lipofectamine 3000 Transfection Kit (Invitrogen) following the protocol recommended by the manufacturer. Transfected cells were selected by puromycin (3 µg/mL).

### 2.5. Cell Lysate Preparation and Immunoblotting

This has been performed as previously described [22]. The list of used antibodies is shown in Supplementary Table S2.

### 2.6. Cell Invasion, Migration, and Proliferation Assays

Cell invasion, migration and proliferation were evaluated using the Real-Time Cell Analyzer—Dual Plat (RTCA-DP) xCELLigence System as per the manufacturer guidelines. In brief, 100 μL of serum-free medium containing 10,000 cells were loaded into the upper wells of CIM-Plate (AGILENT, Santa Clara, CA, USA), while 200 μL of complete media were added to the lower chamber. 20 μL of Matrigel (BD Biosciences, Franklin Lakes, NJ, USA) basement membrane matrix was used to coat the microporous membrane for invasion or without for migration. For the proliferation assay, E-plate (AGILENT) was used and 100 μL of complete medium containing 10,000 cells was loaded into each well. The plates were incubated for 30 min and then was inserted into the RTCA-DP instrument inside a humidified, 37 °C, 5% CO_2_ incubator. The obtained data were analyzed by RTCA software (1.2.1).

### 2.7. 3D Spheroid Assay

Cells were seeded in an ultra-low attachment 96-well plate in stem cell culture medium (171 medium supplemented with 1% ABM, 2% B-27, 20 ng/mL EGF, 500 ng/mL HC, 4% FBS and 5 μg/mL insulin) at a density of 2000 cells/well. Spheres were allowed to form for at least 7 days in a humidified, 37°C, 5% CO_2_ incubator. Spheres larger than 50 µm in diameter were counted using inverted microscope (FLoid Cell Imaging Station from OPTICA, Washington, DC, USA).

### 2.8. Three-Dimensional Cell Culture

MCF-7 cells (3 × 10^6^) were seeded on Biotek 3D Insert scaffold in 6-well plate according to the manufacturer’s instructions. Briefly, cells were re-suspended in 300 μL complete medium and carefully placed onto the center of the scaffold surface. After 3 h, 1700 μL of fresh medium was added into each well. Cell growth was monitored for 2 days to allow for 3D structure formation. The medium was changed, and cells were treated for 24 h. Cells were harvested from the scaffolds for further analysis.

### 2.9. Serum-Free Conditioned Media Preparation

Newly seeded cells were cultured in serum-free medium (0.5% FBS). After 24 h, media were collected and briefly centrifuged. The resulting serum-free conditioned medium (SFCM) was frozen at −80 °C until use.

### 2.10. ELISA Assays

Serum-free conditioned media were collected to be used for ELISA assay after normalizing to cell number. All experiments were performed according to the manufacturer’s instructions. Human Osteoprotegerin/TNFRSF11B DuoSet ELISA (DY805), Human CXCL12/SDF-1 alpha Quantikine ELISA Kit (SSA00), Human TGF-β1 Quantikine ELISA Kit (DB100B), and Human IL-6 Quantikine ELISA Kit (9S6050) were purchased from R&D Systems, Minneapolis, MN, USA.

### 2.11. Human Cytokine Antibody Array

Human Cytokine Array C5 kit (RayBiotech, Georgia, GA, USA) was used according to the manufacturer’s instructions.

### 2.12. Orthotopic Tumor Xenografts

Animal experiments were approved by the KFSH&RC Institutional Animal Care and Use Committee (ACUC) and were conducted according to relevant national and international guidelines. Orthotopic breast tumor xenografts were created by co-injecting MDA-MB-231 breast cancer cells (2.10⁶) with (1.5.10⁶) TCF64-OPG-ORF (TCF64-ORF-T) or TCF64-Ctl cells (TCF64-Ctl-T) under the nipple of each mouse (*n* = 6). Tumor volumes were measured using a caliper and were calculated according to the prolate ellipsoid formula: Tumor volume = length × (width)^2^ × 0.5. Tumors were surgically retrieved and were snap-frozen in liquid nitrogen.

### 2.13. Statistical Analysis and Quantification

Statistical analysis was performed using student’s *t*-test and Ordinary one-way ANOVA using Excel and GraphPad Prism software. *p*-values less than 0.05 were considered as statistically significant. Quantification was performed using ImageJ.

## 3. Results

### 3.1. OPG Is Upregulated in Active Breast Cancer-Associated Fibroblasts

**Figure 1:**
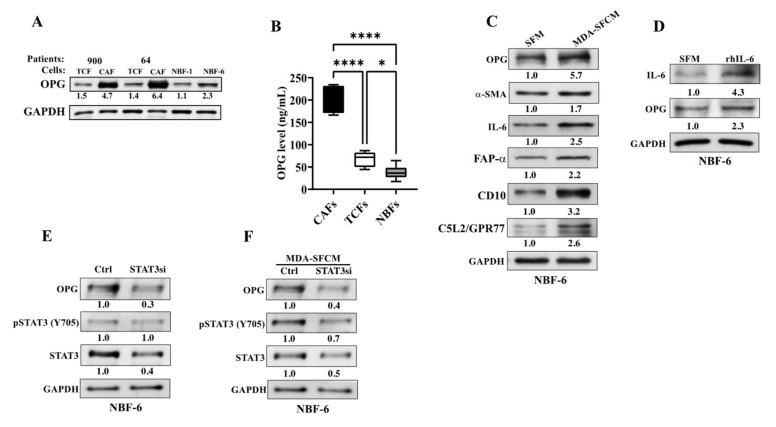
Breast cancer cells and IL-6 upregulate OPG in CAFs through the IL-6/STAT3 pathway. (**A**) Whole cell lysates were prepared from the indicated cells and were used for immunoblotting analysis. The numbers below the bands represent OPG relative expression after correction against the internal control GAPDH. (**B**) Serum-free conditioned media from CAF-64, CAF-87 (CAFs), TCF-64, TCF-87 (TCFs) and NBF-1, NBF-6, NBF-20, NBF-25 (NBFs) were collected after 24 h of incubation with SFM and the levels of the OPG protein were determined by ELISA. Error bars represent mean ± SEM, *p < 0.05; **** *p* < 0.0001 by Ordinary one-way ANOVA. (**C**) NBF6 cells were cultured either in SFM or in MDA-MB-231 SFCM (MDA-SFCM) for 24 h, and then whole cell lysates were prepared for immunoblotting using specific antibodies for the indicated proteins. (**D**) NBF6 cells were cultured either in SFM or SFM containing 3.5 ng/mL of the rhIL-6 protein for 24 h. Whole cell lysates were prepared for immunoblotting analysis using specific antibodies for the indicated proteins. All values were determined by densitometry relative to GAPDH and presented as fold change relative to the respective controls. (**E**,**F**) NBF6 cells were transfected with STAT3 siRNA (STAT3si) or a scrambled sequence (Ctrl). Ctrl and STAT3si cells were either not treated (**E**) or exposed to MDA-MB-231 SFCM (MDA-SFCM) for 24 h (**F**). Cell lysates were then prepared for immunoblotting using specific antibodies for the indicated proteins. The numbers below the bands represent fold change relative to the control (SFM) after correction against the internal control GAPDH. The level of the phosphorylated-STAT3 was normalized against the non-phosphorylated form of the protein.

To address the role of OPG in breast stromal fibroblasts, the basal level of this protein was first examined in CAFs and their corresponding tumor counterpart fibroblasts (TCFs) isolated from adjacent histologically normal tumor tissues. In addition, two normal breast fibroblasts (NBFs) were used for comparison. CAF/TCF pairs and NBFs were always used simultaneously at similar passages. Whole cell lysates were prepared for immunoblotting analysis and antibodies directed against OPG and GAPDH (used as internal control) were used. Figure 1A shows that the OPG protein level is higher in CAFs compared to their corresponding TCFs, which exhibited a level similar to that observed in NBFs (Figure 1A).

Further, the secreted levels of OPG were also evaluated in CAFs, TCFs and NBFs. To this end, serum-free conditioned media (SFCM) from CAF-64, CAF-87 (CAFs), TCF-64, TCF-87 (TCFs) and NBF-6, NBF-1, NBF-20, NBF-25 (NBFs) were collected after 24 h of incubation in the presence of serum-free medium (SFM), and then the level of the secreted OPG protein was determined by ELISA. Figure 1B shows that the OPG secretion level significantly differs between CAFs, TCFs and NBFs, with the highest level observed in CAFs relative to their TCFs, while NBFs secrete the lowest level of OPG. These results confirm the immunoblotting analysis data shown in Figure 1A, and indicate that OPG is upregulated in the active breast cancer-associated fibroblasts. 

### 3.2. Breast Cancer Cells Upregulate OPG in Breast Stromal Fibroblasts in a Paracrine Manner through the IL-6/STAT3 Pathway

The high level of OPG in CAFs could be due to the presence of these cells in close vicinity with cancer cells. To test this hypothesis, NBF-6 cells were treated with SFCM collected from MDA-MB-231 (MDA-SFCM) or with SFM used as a negative control for 24 h, and then whole cell lysates were prepared and used for immunoblotting analysis. The obtained results show increase in the level of the CAF biomarkers, α-SMA and IL-6 accompanied with a 5.7-fold increase in the level of the OPG protein (Figure 1C). Since breast cancer cells mediate their paracrine pro-carcinogenic effects through the IL-6/STAT3 pathway [23], we tested the effect of exogenous recombinant human IL-6 (rIL-6). Therefore, NBF-6 cells were treated with SFM containing or not containing rIL-6 for 24 h, and then the level of OPG was assessed by immunoblotting. As expected, rIL-6 upregulated IL-6 and also OPG (2.3-fold) (Figure 1D).

We have previously shown that OPG is under the control of the STAT3 transcription factor in breast cancer cells [6]. To test the possible role of STAT3 in OPG upregulation in breast stromal fibroblasts, STAT3 was knocked-down in NBF-6 cells with a specific STAT3-siRNA (STAT3si), while a scrambled sequence was used as the control (Ctrl). The immunoblotting analysis confirmed the downregulation of STAT3 in NBF-6 STAT3si cells as compared to their controls (Figure 1E). STAT3 knockdown was accompanied with a 3.3-fold reduction in the OPG protein level (Figure 1F). This confirms that STAT3 positively controls the expression of OPG in breast stromal fibroblasts. Next, STAT3si and Ctrl cells were treated with MDA-SFCM for 24 h. Interestingly, STAT3 knockdown abolished MDA-SFCM-dependent upregulation of OPG in NBF-6 cells (Figure 1F). This indicates that breast cancer cells upregulate OPG in breast stromal fibroblasts through the IL-6 protein and its canonical STAT3 pathway. 

### 3.3. Ectopic Expression of OPG Activates Breast Stromal Fibroblasts through the NF-κB/STAT3/AUF1 Signaling Pathway

**Figure 2:**
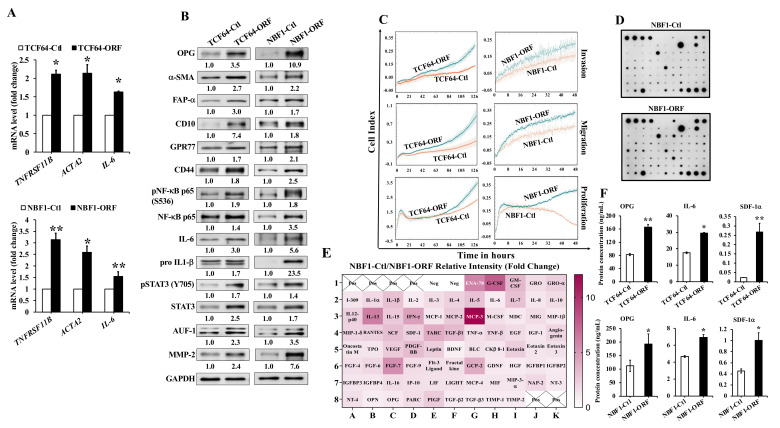
Ectopic expression of OPG activates breast stromal fibroblasts.TCF-64 and NBF-1 cells were transfected with a plasmid expressing OPG-ORF (TCF64-ORF and NBF1-ORF) or an empty vector (TCF64-Ctl and NBF1-Ctl), respectively. (**A**) Total RNA was extracted from the indicated cells, and the mRNA levels of the indicated genes were assessed by qRT-PCR using specific primers for the indicated genes. Error bars represent mean ± S.D. * *p* < 0.05, ** *p* < 0.005. (**B**) Whole cell lysates were prepared from the indicated cells, and then were used for immunoblotting analysis using specific antibodies against the indicated proteins. The numbers below the bands represent fold change relative to the control (SFM) after correction against the internal control GAPDH. The levels of phosphorylated proteins were normalized against the total amount of their relative non-phosphorylated forms. (**C**) Cell invasion, migration, and proliferation abilities were assessed using the Real-Time Cell Analyzer-Dual Plate (RTCA-DP) xCELLigence System. Data are representative of different experiments performed in triplicate. (**D**) SFCM from the indicated cells were collected after 24 h and were applied on the RayBiotech Human Cytokine Array C5. The spots correspond to cytokines. (**E**) The intensities of the spots in the cytokine array were quantified by densitometric analysis and normalized to the density of the positive controls of each membrane (POS) and were presented as fold change in the single gradient heatmap. (**F**) SFCM from the indicated cells were collected after 24 h and the levels of the indicated proteins were determined by ELISA and presented in the respective histograms. Error bars indicate mean ± S.D. (*n* = 3). * *p* < 0.05, ** *p* < 0.005.

To elucidate the role of OPG upregulation in the activation of breast stromal fibroblasts, a plasmid bearing the OPG-open reading frame (ORF) was introduced into TCF-64 and NBF-1 cells (TCF64-ORF and NBF1-ORF, respectively), while the corresponding empty vector was used as a control (TCF64-Ctl and NBF1-Ctl, respectively). As expected, the mRNA level of the OPG coding gene (TNFRSF11B) was increased in TCF64-ORF and NBF1-ORF as compared to their respective controls (Figure 2A). This increase was accompanied with a significant upregulation of ACTA2 (α-SMA) and IL-6 in both cell cultures (Figure 2A). The OPG protein level was also increased 3.5-fold and 10.9-fold in TCF64-ORF and NBF1-ORF as compared to their respective controls (Figure 2B). OPG upregulation was accompanied in both cell cultures with a strong increase in the level of the active fibroblast biomarkers α-SMA, FAP-α, CD10, and GPR77 relative to the controls (Figure 2B). CD44, which has been shown to be upregulated in active stromal fibroblasts [24], was also upregulated in OPG-expressing cells (Figure 2B). This indicates that OPG upregulation can lead to different subtypes of active breast stromal fibroblasts. 

To confirm OPG-dependent activation of BSFs, the effect of OPG upregulation on the invasion, migration, and proliferation abilities of TCF and NBF cells were evaluated using the Real-Time Cell Analyzer-Dual Plate (RTCA-DP) xCELLigence System. The invasion, migration, and proliferation abilities of TCF64-ORF and NBF1-ORF cells were higher than their respective controls (Figure 2C). The increase in the invasiveness ability was accompanied with clear upregulation of the pro-invasive MMP-2 protein (Figure 2B). 

Next, SFCM collected from NBF1-ORF or NBF1-Ctl cells were applied on the Human Cytokine Antibody Array as shown in Figure 2D. The cytokine array analysis showed an increase in the pro-carcinogenic cytokines (more than 3-fold increase); MCP-3, G-CSF, IL-13, ENA-78, FGF-7, IFN-γ, TARC, GCP-2, GM-CSF, TGF-β1, PDGF-BB, SCF, IL-1β, IL-7, and IL-5 in the NBF1-ORF cells as compared to the controls (Figure 2E). ELISA showed that the secreted levels of the OPG protein were increased 2.0- and 1.7-fold in TCF64-ORF and NBF1-ORF cells as compared to their respective controls (Figure 2F). OPG upregulation also increased the secretion levels of the IL-6 and SDF-1α proteins (Figure 2F). These results indicate that OPG upregulation in NBFs and TCFs increases the expression/secretion levels of several cancer-promoting proteins/cytokines. 

To understand the molecular basis of the OPG-dependent activation of NBFs, the effect of OPG upregulation was first tested on the important NF-κB pathway, a target of the OPG/RANKL signaling pathway [16]. Interestingly, OPG upregulation in both cell cultures promoted the activation/phosphorylation of NF-κB (p65) at Ser536 (Figure 2B). This was confirmed by showing the upregulation of IL-6 at both the protein and mRNA levels, and the non-active form of IL-1β, the pro IL-1β (Figure 2B). The presence of functional interaction between the NF-κB and STAT3 pathways prompted us to check the effect of OPG upregulation on this transcription factor. Figure 2B shows OPG-dependent activation/phosphorylation of STAT3 at Tyr705 a site that has been found to be phosphorylated in CAFs and in IL-6-dependent activation of breast stromal fibroblasts [23]. STAT3 regulates IL-6 at the post-transcriptional level through AUF1, which controls also other CAF markers such as α-SMA and SDF-1 [23]. Interestingly, OPG upregulation increased the expression of AUF1 relative to the control cells (Figure 2B). This indicates that OPG could activate BSFs through the activation of STAT3 and the consequent upregulation of AUF1, a major regulator of several active CAF biomarkers. 

### 3.4. OPG Upregulation Promotes the Paracrine Pro-EMT Effect of Breast Stromal Fibroblasts in an IL-6-Dependent Manner

**Figure 3:**
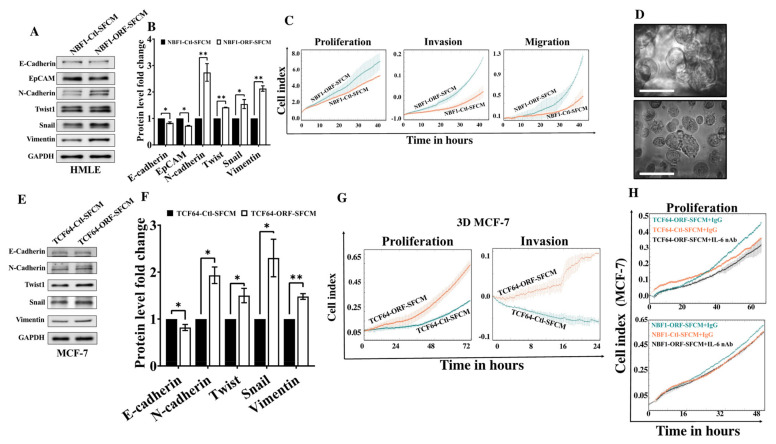
OPG upregulation in NBFs promotes the EMT process in BC cells in a paracrine fashion. (**A**) Immortalized human mammary epithelial cells (HMLE) were treated for 24 h with SFCM collected from NBF1-ORF-SFCM or NBF1-Ctl-SFCM. Whole cell lysates were prepared, and immunoblotting analysis was performed using antibodies against the indicated proteins. (**B**) Quantification of the immunoblots shown in (**A**) was performed by densitometry relative to GAPDH and presented as fold change relative to the control. Error bars represent mean ± SD (*n* = 3). * *p* < 0.05, ** *p* < 0.005. (**C**) The proliferation, invasion, and migration capabilities of HMLE cells treated with the indicated SFCM were assessed using the RTCA-DP xCELLigence system. Data are representative of different experiments performed in triplicate. (**D**) MCF-7 cells were seeded on 3D Insert™ scaffold in a 6-well cell culture plate for 48 h. Three-dimensional (3D) aggregates and rounded cell structures on PS scaffold pores are shown. Scale bar = 50 µm. (**E**) 3D MCF-7 cells were treated for 24 h with SFCM collected from TCF64-ORF-SFCM and TCF64-Ctl-SFCM. Whole cell lysates were prepared, and immunoblotting analysis was performed using antibodies against the indicated proteins. (**F**) Quantification of the immunoblots shown in (**E**) relative to GAPDH and presented as fold change relative to the control. Error bars represent mean ± SD (*n* = 3). * *p* < 0.05, ** *p* < 0.005. (**G**) The proliferation, invasion capabilities of the indicated cells were assessed using the RTCA-DP xCELLigence system. (**H**) The proliferation capabilities of the indicated cells were assessed using the RTCA-DP xCELLigence system. Data are representative of different experiments performed in triplicate.

In order to demonstrate the active status of BSFs that express high level of OPG, we tested their paracrine effects on normal breast luminal cells (cancer initiation) as well as on breast cancer cells (cancer promotion). Therefore, SFCM from NBF1-ORF and NBF1-Ctl cells were used to treat HMLE cells for 24 h. The immunoblotting analysis shows a slight but significant decrease in the epithelial markers, E-cadherin and EpCAM, in NBF1-ORF-SFCM treated HMLE cells as compared to the controls (Figure 3A,B). Interestingly, HMLE cells treated with NBF1-ORF-SFCM showed a clear increase in the mesenchymal markers N-cadherin, Twist1, Snail, and Vimentin relative to their levels in the control cells (Figure 3A,B). To confirm this epithelial-to-mesenchymal transition (EMT) induction, the Real-Time Cell Analyzer was used to show that the proliferation, invasion, and migration capacities of HMLE cells were increased in the presence of NBF1-ORF-SFCM as compared to their controls (Figure 3C). This indicates that OPG upregulation in BSFs enhances their paracrine abilities to induce the EMT process in breast epithelial cells, with a marked effect on the mesenchymal biomarkers as compared to the epithelial ones. 

Next, the paracrine effects of OPG upregulation in BSFs were tested on breast cancer cells grown in 3D. To this end, MCF-7 cells were seeded on the 3D Insert™ scaffold and monitored for 48 h until 3D structures were formed as shown in Figure 3D. MCF-7 cells grown in 3D were treated for 24 h with SFCM collected from TCF64-ORF and TCF64-Ctl, and then whole cell lysates were prepared for immunoblotting. Figure 3E and F shows that TCF64-ORF-SFCM slightly decreased the level of the epithelial marker E-cadherin and upregulated the mesenchymal markers N-cadherin, Twist, Snail, and Vimentin relative to their levels in the control cells. Furthermore, TCF64-ORF-SFCM enhanced the proliferative and the migratory potential of MCF-7 cells grown in 3D culture (Figure 3G). This shows that breast stromal fibroblasts that express a high level of OPG can promote EMT in breast cancer spheroids. 

Recently, OPG was found to play a role in the NBF-related paracrine inhibition of breast carcinogenesis and this effect was suppressed by the addition of the IL-6 protein [6]. Therefore, the paracrine pro-carcinogenic effect of OPG expressing cells could be due to the upregulation of IL-6 rather than to an increase in the secreted level of OPG. To test this hypothesis, IL-6 was neutralized in NBF1-ORF-SFCM as well as in TCF64-ORF-SFCM using a specific neutralizing anti-IL-6 antibody (1 μg/mL), while IgG was used as a negative control. These SFCM were used to treat MCF-7 cells for 24 h, and then cellular proliferation was analyzed. Interestingly, the proliferation of MCF-7 cells treated with TCF64-ORF-SFCM+IL-6 nAb was reduced to a rate similar to that of control cells (Figure 3H). Similar result was obtained for MCF-7 cells treated with NBF1-ORF-SFCM+IL-6 nAb. These results indicate that IL-6 specific inhibition suppresses the paracrine pro-proliferative effect of OPG expressing BSFs (Figure 3H). Thus, the paracrine pro-carcinogenic effects of OPG-expressing BSFs are mediated through pro-carcinogenic proteins, such as IL-6. 

### 3.5. OPG Upregulation Promotes the Paracrine Pro-Stemness Effect of Breast Stromal Fibroblasts in an IL-6-Dependent Manner

**Figure 4:**
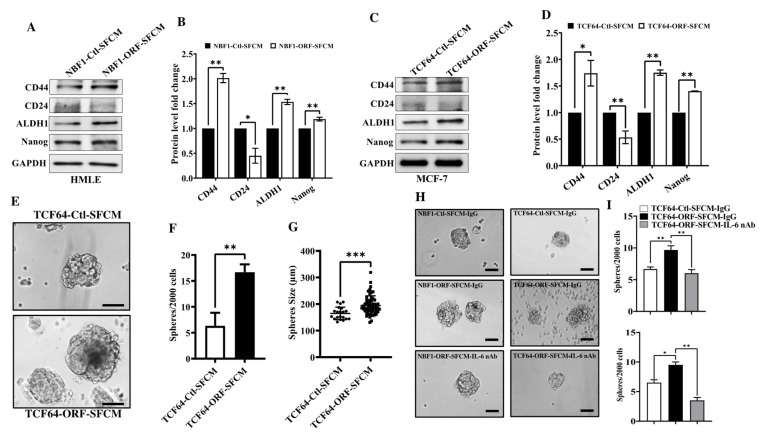
OPG upregulation in NBFs promotes stemness in BC cells in a paracrine manner. (**A**–**D**) Figure legends are as in Figure 3A,B. (**E**) MCF-7 cells were treated with the indicated SFCM, and then were seeded in ultra-low attachment 96 well plate in stem cell culture medium at a density of 2000 cells/well. Spheres were allowed to form for 10 days, and representative ones are shown. Scale bar = 100 µm. (**F**) Spheres (˃100 µm) of MCF-7 treated with the indicated SFCM were counted under an inverted microscope and presented as histogram. Error bars indicate mean ± SD (*n* = 3). ** *p* < 0.005. (**G**) MCF-7 spheres (˃100 µm) treated with the indicated SFCM were measured using ImageJ. Error bars indicate mean ± SD (*n* = 3). *** *p* < 0.0005. (**H**) MCF-7 cells treated with the indicated SFCM were seeded in an ultra-low attachment 96 well plate in stem cell culture medium at a density of 2000 cells/well. Representative MCF-7 spheres were imaged using an inverted microscope (FLoid Cell Imaging Station). Scale bar = 100 µm. (**I**) Spheres (˃100 µm) were counted and the numbers were presented as histogram. Error bars indicate mean ± SD (*n* = 3). * *p* < 0.05, ** *p* < 0.005.

In addition to EMT, TCF64-ORF-SFCM also increased the expression level of the stemness markers CD44, ALDH1 and Nanog, while there was a clear decrease in the level of CD24 as compared to their levels in the control cells (Figure 4A,B). Similar results were obtained for MCF-7 cells grown in 3D and treated with TCF64-ORF-SFCM or TCF64-Ctl-SFCM (Figure 4C,D).

To confirm the induction of cancer stem cells features, we tested the effect of TCF64-ORF-SFCM on sphere formation ability of MCF-7 cells. Therefore, MCF-7 cells treated with TCF64-ORF-SFCM or TCF64-Ctl-SFCM for 24 h were seeded in ultra-low attachment 96 well plate in stem cell culture medium at a density of 2000 cells/well. Primary mammopheres were allowed to form for 10 days (Figure 4E). Interestingly, the number of spheres larger than 100 µm in diameter in MCF-7 cells treated with TCF64-ORF-SFCM was significantly higher than the number of spheres formed in MCF-7 treated with TCF64-Ctl-SFCM (Figure 4F). Moreover, sphere sizes of MCF-7 treated with TCF64-ORF-SFCM was significantly larger than the spheres formed in the control cells as shown in Figure 4G. These results indicate that OPG upregulation in BSFs induces their paracrine pro-carcinogenic effects through the promotion of EMT and stemness.

It has also been shown that OPG suppresses the stemness characteristics of BC cells [6]. This means that the OPG-dependent paracrine induction of stemness in BC cells could be IL-6-dependent. To confirm this possibility, IL-6 was neutralized in TCF64-ORF-SFCM and NBF1-ORF-SFCM before treatment of MCF-7 and the assessment of sphere formation ability. Subsequently, cells were seeded in ultra-low attachment 96-well plates in stem cell culture medium at a density of 2000 cells/well, and primary mammopheres were allowed to form for 15 days (Figure 4H). Interestingly, the number of tumorspheres formed in MCF-7 cells treated with NBF1-ORF-SFCM+IL-6 nAb was significantly lower than the number of spheres formed in MCF-7 treated with NBF1-ORF-SFCM+IgG as shown in Figure 4H and 4I. A similar result was obtained for MCF-7 cells treated with TCF64-ORF-SFCM+IL-6 nAb (Figure 4H,I). These results indicate that the paracrine pro-stemness effect of OPG expressing BSFs is IL-6-dependent. 

### 3.6. OPG Upregulation in Breast Stromal Fibroblasts Enhances Their Paracrine Pro-Carcinogenic Effects In Vivo

**Figure 5:**
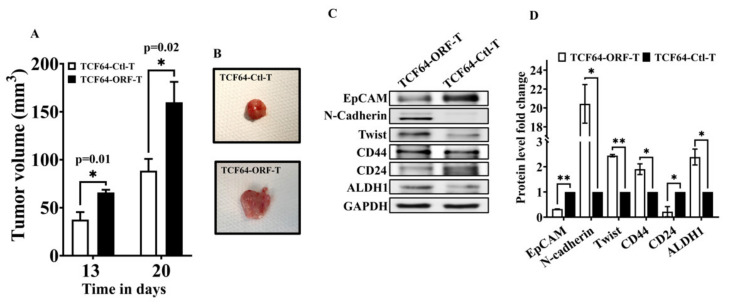
OPG upregulation in breast stromal fibroblasts promotes orthotopic tumor growth. Breast cancer orthotopic xenografts were created in female nude mice by co-injecting MDA-MB-231 cells (2.10⁶) with 1.5 × 10⁶ TCF64-OPG-ORF (TCF64-ORF-T) or TCF64-Ctl cells (TCF64-Ctl-T) under the nipple of each mouse (*n* = 6). (**A**) Tumor volumes were measured at the indicated periods of time and were depicted as histogram. Error bars represent mean values (±SEM). (**B**) Pictures of one excised tumor from each group are shown. (**C**) Whole cell lysates were prepared from the excised tumors and were utilized for immunoblotting analysis using antibodies against the indicated proteins. (**D**) Quantification of the immunoblots shown in (**C**) was performed by densitometry relative to GAPDH and presented as fold change relative to the control. Error bars represent mean ± SEM (*n* = 3). * *p* < 0.05, ** *p* < 0.005.

Next, we sought to assess the paracrine pro-carcinogenic effects of BSFs expressing high level of OPG in vivo. To this end, nude mice were randomized into two groups and orthotopic breast tumor xenografts were created by co-injecting MDA-MB-231 breast cancer cells (2 × 10⁶) with 1.5 × 10⁶ TCF64-OPG-ORF (TCF64-ORF-T) or TCF64-Ctl cells (TCF64-Ctl-T) under the nipple of each mouse (*n* = 6). Interestingly, tumors bearing TCF64-OPG-ORF cells (TCF64-ORF-T) grew significantly faster relative to those having control cells (TCF64-Ctl-T) (Figure 5A). Next, tumors were excised and a picture of one tumor from each group was depicted showing the bigger size of the tumor bearing TCF64-OPG-ORF cells as compared to the control (Figure 5B). This indicates that OPG upregulation in BSFs enhance their ability to promote tumor growth in vivo. Next, whole cell lysates were prepared and were used for immunoblotting analysis. The level of the epithelial marker EpCAM was significantly lower in TCF64-ORF-T as compared to TCF64-Ctl-T (Figure 5C,D). On the other hand, the levels of the mesenchymal markers N-cadherin and Twist1 were much higher in TCF64-ORF-T as compared to the control (Figure 5C,D). Additionally, TCF64-ORF-T expressed higher levels of the stemness markers, CD44 and ALDH1, but lower level of CD24 as compared to the control tumor TCF64-Ctl-T (Figure 5C,D). Therefore, TCF64-OPG-ORF cells induced EMT and stemness in breast cancer cells in vivo as well. These results indicate that OPG upregulation in BSFs enhances their ability to promote breast carcinogenesis in orthotopic tumor xenografts.

### 3.7. OPG Downregulation Suppresses Active Breast Stromal Fibroblasts

**Figure 6:**
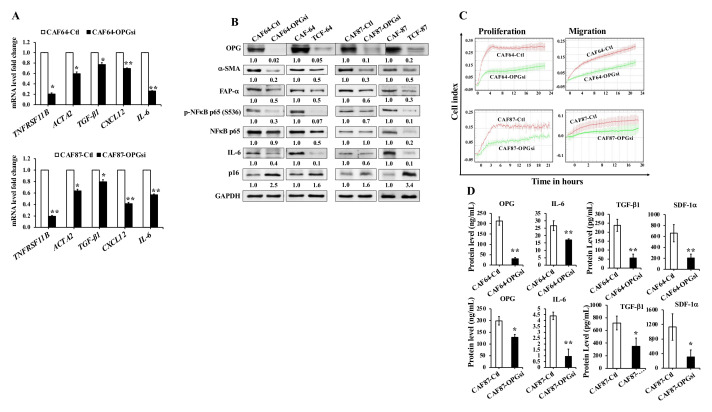
OPG downregulation suppresses active CAFs. CAF-64 and CAF-87 cells were transfected with specific OPG-siRNA (CAF64-OPGsi and CAF87-OPGsi) and a scrambled sequence was used as a control (CAF64-Ctl and CAF87-Ctl), respectively. (**A**) Total RNA was extracted from the indicated cells, and the mRNA levels of the indicated genes were assessed by qRT-PCR using specific primers for the indicated genes. Error bars represent mean ± S.D. * *p* < 0.05, ** *p* < 0.005. (**B**) Whole cell lysates were prepared from the indicated cells, and then were used for immunoblotting analysis using specific antibodies against the indicated proteins. The numbers below the bands indicate band intensity normalized to GAPDH, while phospho-proteins were further normalized to the total protein and presented as fold change as compared to the control. (**C**) Exponentially growing cells were seeded in E-plate for proliferation and CIM-plate for migration, and cell proliferation/migration were assessed using the Real-Time Cell Analyzer-Dual Plate (RTCA-DP) xCELLigence System. Data are representative of different experiments performed in triplicate. (**D**) SFCM from the indicated cells were collected after 24 h and the levels of the indicated proteins were determined by ELISA and presented in the respective histograms. Error bars indicate mean ± SEM (*n* = 3). * *p* < 0.05, ** *p* < 0.005.

To further show the role of OPG in the activation of breast stromal fibroblasts (BSFs), OPG was knocked-down using specific OPG siRNA in CAF-64 and CAF-87 cells, while a scrambled sequence was used as a control. Significant knockdown of the OPG gene (*TNFRSF11B*) (5 fold) was observed at the mRNA level for both CAF64-OPGsi and CAF87-OPGsi as compared to their respective control cells (Figure 6A). This decrease was accompanied with a decrease in the mRNA levels of the active fibroblast biomarkers *ACTA2* (α-SMA), *TGF-β1*, *CXCL12* (SDF-1), and *IL-6* in OPG-defective cells (CAF64-OPGsi and CAF87-OPGsi) as compared to their corresponding controls. Figure 6B shows that the OPG protein level was also declined 50-fold and 10-fold in CAF64-OPGsi and CAF87-OPGsi as compared to their respective controls. This was accompanied with a strong decrease in the levels of the important active fibroblast biomarkers, α-SMA and FAP-α, to a level similar to that observed in TCF cells (TCF-64 and TCF-87) (Figure 6B). While silencing OPG in both cell cultures did not alter the basal expression of NF-κB, the level of the phospho-NF-κB ^S536^ was decreased, in addition to a decrease in its downstream target, IL-6 (Figure 6B). On the other hand, OPG downregulation increased the level of the tumor suppressor protein p16 (2.5- and 1.6-fold) in CAF64-OPGsi and CAF87-OPGsi, respectively (Figure 6B). Therefore, OPG downregulation normalizes the expression level of the biomarkers of active BSFs. Furthermore, a clear decrease was noticed in the proliferation and the migration abilities of CAF64-OPGsi and CAF87-OPGsi cells as compared to their respective controls (Figure 6C). This indicates that OPG downregulation decreases the proliferation and the migration abilities of active CAFs.

Additionally, we assessed the effect of OPG downregulation on the secretion of some important cytokines by ELISA. Figure 6D shows that the secreted levels of the OPG protein were decreased 7.0- and 1.5-fold in CAF64-OPGsi and CAF87-OPGsi, respectively. OPG downregulation also reduced the secretion levels of the IL-6, TGF-β1 and SDF-1α proteins (Figure 6D). These results indicate that OPG knockdown suppresses the expression/secretion of the cancer-promoting proteins in CAF cells.

### 3.8. OPG Downregulation Suppresses the Paracrine Pro-Carcinogenic Effects of Active CAFs

**Figure 7:**
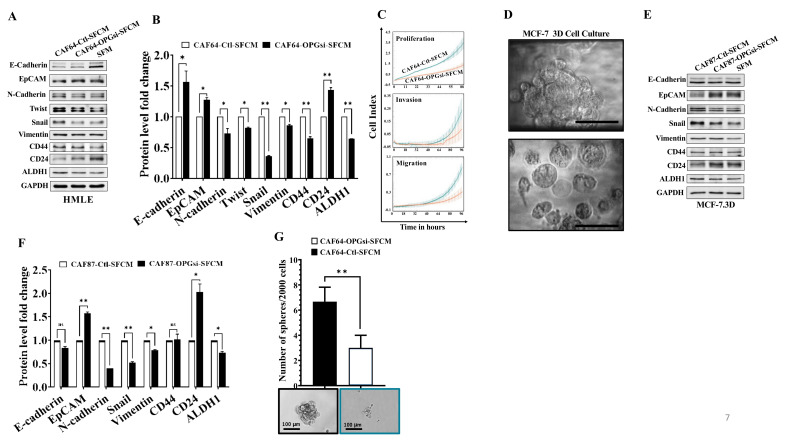
OPG downregulation suppresses the paracrine pro-carcinogenic effects of active breast stromal fibroblasts. (**A**) Cells were treated with SFCM collected from CAF64-OPGsi or CAF64-Ctl for 24 h. Whole cell lysates were prepared, and immunoblotting analysis was performed using antibodies against the indicated proteins. (**B**) Quantification of the immunoblots shown in (**A**) were determined by densitometry relative to GAPDH and presented as fold change relative to the control. Error bars represent mean ± SD (*n* = 3). * *p* < 0.05, ** *p* < 0.005. (**C**) The proliferation, invasion, and migration capabilities of HMLE cells treated with the indicated SFCM were assessed using the RTCA-DP xCELLigence system. Data are representative of different experiments performed in triplicates. (**D**) MCF-7 cells were seeded on 3D Insert™ scaffold in a 6-well cell culture plate for 48 h. Three-dimensional (3D) aggregates and rounded cell structures on PS scaffold pores are shown. Scale bar = 50 µm. (**E**) MCF-7 spheroids were treated for 24 h with SFCM collected from CAF87-OPGsi and CAF87-Ctl cells. Whole cell lysates were prepared, and immunoblotting analysis was performed using antibodies against the indicated proteins. (**F**) Quantification of the immunoblots shown in (**E**) relative to GAPDH and presented as fold change relative to the control. Error bars represent mean ± SD (*n* = 3). * *p* < 0.05, ** *p* < 0.005.

Next, we sought to investigate the paracrine effects of OPG downregulation in active CAFs on normal breast as well cancerous epithelial cells. To this end, SFCM collected from CAF64-OPGsi and CAF64-Ctl were first used to treat HMLE cells for 24 h. The immunoblotting analysis shows that CAF64-OPGsi-SFCM increased the level of the epithelial markers E-cadherin and EpCAM, while it downregulated the mesenchymal markers *N*-cadherin, Twist, Snail, and Vimentin, relative to their levels in the control cells (CAF64-Ctl-SFCM) (Figure 7A,B). To confirm this pro-EMT inhibitory effect, we showed that the proliferation, invasion, and migration capacities of HMLE cells were lower in the presence of CAF64-OPGsi-SFCM as compared to their controls. This indicates that OPG downregulation in active CAFs reduces their paracrine pro-carcinogenic EMT process in breast epithelial cells. Moreover, CAF64-OPGsi-SFCM decreased the expression of the stemness markers CD44 and ALDH1, while it increased the level of CD24, as compared to their levels in the control cells (Figure 7A,B). These results show that OPG downregulation in active CAFs suppresses their paracrine pro-carcinogenic effects.

Moreover, we decided to use 3D cell culture in order to mimic the in vivo cellular structure. Therefore, MCF-7 were seeded on the 3D Insert™ scaffold and monitored for 48 h until 3D structures were formed as shown in Figure 7D. MCF-7 spheroids were treated for 24 h with SFCM collected from CAF87-OPGsi and CAF87-Ctl, and then whole cell lysates were prepared for the immunoblotting assay. Figure 7E,F shows that CAF87-OPGsi-SFCM did not affect the protein level of the epithelial marker E-Cadherin, while there was a significant increase in the epithelial marker EpCAM. On the other hand, CAF87-OPGsi-SFCM downregulated the mesenchymal markers N-cadherin, Snail, and Vimentin relative to their levels in the control cells (Figure 7E,F). Furthermore, while CAF87-OPGsi-SFCM didn’t affect the expression of CD44, there was a significant decrease in the expression of ALDH1 and increase in the level of CD24 protein as compared with their respective levels in the control cells (Figure 7E,F). These results were confirmed by showing that the self-renewal capacity and the ability of MCF-7 cells to form tumorspheres was significantly reduced in the presence of CAF87-OPGsi-SFCM relative to the controls (Figure 7G). These results indicate that OPG downregulation in active CAFs suppresses their ability to promote EMT and stemness in normal and cancer epithelial cells.

### 3.9. Human Recombinant OPG Suppresses the Paracrine Pro-Carcinogenic Effects of Active CAFs

**Figure 8:**
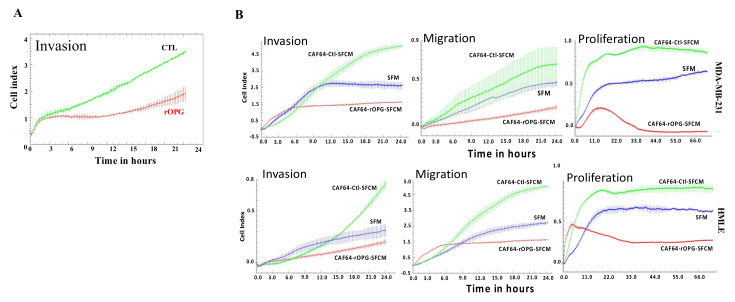
rOPG normalizes the active features of CAFs and suppresses their paracrine pro-carcinogenic effects. (**A**) CAF-64 cells were either sham-treated (CTL) or challenged with rOPG (10 ng/mL), and then the invasion capability of cells was assessed using the RTCA-DP xCELLigence system. Data are representative of different experiments performed in triplicates. (**B**) The invasion/migration and proliferation capabilities of HMLE and MDA-MB-231 cells treated as indicated were assessed using the RTCA-DP xCELLigence system. Data are representative of different experiments performed in triplicates.

We have recently shown that the human recombinant OPG protein (rOPG) inhibits the invasive capacity of breast cancer cells [6]. Thus, we sought to study the effect of rOPG on active breast stromal fibroblasts and the possible normalization of these cells. To this end, CAF-64 cells were either sham-treated of exposed to rOPG 10 ng/mL for 24 h. Figure 8A shows strong inhibition of the invasiveness capacity of CAF-64 cells when exposed to rOPG. In addition, we showed that SFCM from rOPG-treated CAF-64 cells suppressed the paracrine pro-invasive/migratory and proliferative effects of control cells on breast epithelial cells as well as MDA-MB-231 cells (Figure 8B). These results indicate that rOPG can normalize the autocrine and paracrine features of active breast cancer-associated fibroblasts.

## 4. Discussion

In the present study, we sought to delineate the role of OPG in BSFs. We have first shown that OPG is highly expressed in CAFs as compared to their adjacent TCFs or normal breast fibroblasts. This suggests that breast cancer cells can enhance the expression of OPG in the surrounding stromal fibroblasts. Indeed, triple-negative BC cells upregulated OPG in BSFs in an IL-6-dependent manner through the activation of the STAT3 pathway. Similarly, rhIL-6 was found to increase the OPG level in vascular smooth muscle cells (VSMCs) [25]. In addition to IL-6, TGF-b1 and IL-1b have been found to enhance the expression and secretion levels of OPG in mouse stromal cells and breast cancer cell lines, respectively [26,27]. Thus, the expression of OPG can be modulated in response to various cytokines. Furthermore, we showed that IL-6-related upregulation of OPG is STAT3-dependent. Similarly, it has been recently shown that STAT3 positively controls OPG expression in BC cells [6]. Since STAT3 is persistently active in active fibroblasts, it might be responsible for the persistent expression of OPG in these cells.

To elucidate the effect of OPG upregulation on BSFs, the expression of the gene was increased through ectopic expression in TCF and NBF cells. This led to the activation of these normal fibroblasts and increases in their paracrine pro-carcinogenic effects. Indeed, OPG upregulation enhanced the expression of the major biomarker of active fibroblasts, a-SMA. Consistent with this, it has been previously shown that OPG positively regulates a-SMA in VSMC [28]. Likewise, OPG upregulation enhanced the expression of other active fibroblast biomarkers, namely, FAP-a, CD10, GPR77, and CD44. While FAP-a-expressing active fibroblasts promote breast tumorigenesis and are implicated in an immunosuppressive environment [29], CD10^+^ GPR77^+^ as well as CD44^+^ active breast fibroblasts promote breast cancer stemness and chemoresistance [11,24,30]. Therefore, OPG upregulation in BSFs can induce various subtypes of active fibroblasts with different pro-carcinogenic effects. These findings raised an important question on how OPG controls all these genes and physiological processes. Since these processes are under the control of two important and interconnected pathways, NF-κB and STAT3, we sought to investigate the link between OPG and these two pathways. We found that OPG positively controls NF-κB (p65). Indeed, while OPG upregulation activated p65, OPG knockdown with specific siRNA inhibited this transcription factor. Previous studies have shown that NF-κB positively regulates a-SMA and FAP-a in active fibroblasts [31,32]. Furthermore, NF-κB signaling is responsible for the phenotype and function of CD10^+^GPR77^+^ active fibroblasts [11]. In addition, NF-κB was found to regulate CD44 expression in BC cells [33]. Therefore, OPG could upregulate these CAF biomarkers in BSFs through the activation of NF-κB. We have also shown that OPG positively regulates STAT3 and its downstream effector AUF-1, which stabilizes the expression of the active fibroblast biomarkers, a-SMA, SDF-1, TGF-b1, and IL-6 [23]. Therefore, OPG could also activate BSFs through the STAT3/AUF1 pathway. These findings indicate that endogenous OPG plays a major role in the activation of BSFs through the pro-carcinogenic STAT3/NF-κB signaling, which positively regulates OPG through a feedback loop.

OPG-related activation of BSFs was confirmed by showing that OPG upregulation in BSFs enhanced their proliferation and invasion abilities and the production of the pro-invasive protein MMP-2, as well as the secretion of multiple pro-carcinogenic cytokines, including IL-6, SDF-1 and TGF-b1. The increase in the secretion of these cytokines is one of the main features of active BSFs and play major roles in tumor-promoting function of CAFs. CAF-derived IL-6, SDF-1 and TGF-b1 promote cancer progression through induction of EMT, stemness and angiogenesis in a paracrine manner [3]. Consequently, ectopic expression of OPG significantly enhanced the migration/invasion abilities and increased the expression of several mesenchymal markers (*N*-cadherin, Vimentin, Snail, and Twist1), and reduced the expression of the epithelial markers (E-cadherin and EpCAM) in mammary luminal cells as well as BC cells grown as 3D. Furthermore, OPG expressing fibroblasts promoted stem-like features (CD24^low^/CD44^high^ and ALDH^high^) and the formation of mammospheres in primary luminal cells and BC cells. These results were confirmed in vivo in orthotopic tumor xenografts generated in mice. Therefore, OPG upregulation in breast fibroblasts promotes EMT and stemness as well as tumor growth in a paracrine manner. Interestingly, these pro-carcinogenic effects were shown to be IL-6-dependent. Indeed, anti-IL-6 neutralizing antibody suppressed the paracrine pro-carcinogenic effects of OPG-expressing fibroblasts, which secrete a high level of the anti-carcinogenic factor OPG as well as several other pro-carcinogenic factors such as IL-6 and SDF-1. This indicates that while active breast fibroblasts secrete pro- and anti-carcinogenic cytokines, they promote carcinogenesis due to the dominance of the pro-carcinogenic cytokines. Together, these results show that OPG upregulation activates BSFs and enhances their pro-carcinogenic effects in an IL-6-dependent manner.

On the other hand, OPG downregulation promoted quiescence in active CAF cells and inhibited their paracrine pro-EMT and -stemness/self-renewal processes in human luminal cells as well as in breast cancer cells. These data demonstrated the important role of OPG in the active state of BSFs and their cancer-promoting potential. Therefore, OPG downregulation in active CAFs could constitute an effective CAF-targeting therapeutic approach for breast cancer patients. We showed here that rOPG strongly inhibited their invasive capacity as well as their paracrine pro-carcinogenic effects on primary normal human luminal cells and breast cancer cells. This shows the possible use of rOPG to target both breast cancer cells and their supportive active stromal fibroblasts. It is noteworthy that while only 10 ng/mL is sufficient to normalize active CAFs and inhibits their pro-carcinogenic effects, 1 μg/mL was needed to target breast cancer cells [6]. Therefore, using rOPG at low concentration could be utilized to target tumors or prevent their recurrence and spread through normalization of their microenvironment. The possibility to use rOPG for the treatment of breast tumors and the prevention of bone metastasis was previously proposed and tested with conflicting results. This is mainly due to limited knowledge on the exact role of OPG in breast carcinogenesis, since it has a pro-carcinogenic function when intracellular, and suppresses carcinogenesis when extracellular [16,34]. The fact that secreted OPG and the recombinant form of the protein repress the expression of the endogenous protein through inhibition of the NF-κB pathway could explain some of the observed differences [6].

## 5. Conclusions

In this study, we showed that breast cancer cells can upregulate OPG in stromal fibroblasts in an IL-6/STAT3-dependent manner. This increase in the OPG level activates these adjacent fibroblasts and transforms them to pro-carcinogenic cells, which can promote tumor growth in vivo. Importantly, rOPG can normalize the active features of CAF cells, which suggests the possible use of this cytokine to target active breast stromal fibroblasts.

## Data Availability

Not applicable.

## Supplementary Materials

The following supporting information can be downloaded at: https://www.mdpi.com/article/10.3390/cells11213369/s1, Table S1. List of primers, Table S2. List of antibodies.

## References

[1] Ferlay J., Shin H.R., Bray F., Forman D., Mathers C., Parkin D.M. (2010). Estimates of worldwide burden of cancer in 2008: GLOBOCAN 2008. Int. J. Cancer.

[2] Twigger A.J., Khaled W.T. (2021). Mammary gland development from a single cell ‘omics view. Semin. Cell Dev. Biol..

[3] Sahai E., Astsaturov I., Cukierman E., DeNardo D.G., Egeblad M., Evans R.M., Fearon D., Greten F.R., Hingorani S.R., Hunter T. (2020). A framework for advancing our understanding of cancer-associated fibroblasts. Nat. Rev. Cancer.

[4] Kalluri R. (2016). The biology and function of fibroblasts in cancer. Nat. Rev. Cancer.

[5] Gascard P., Tlsty T.D. (2016). Carcinoma-associated fibroblasts: Orchestrating the composition of malignancy. Genes Dev..

[6] Alraouji N.N., Hendrayani S.F., Ghebeh H., Al-Mohanna F.H., Aboussekhra A. (2021). Osteoprotegerin (OPG) mediates the anti-carcinogenic effects of normal breast fibroblasts and targets cancer stem cells through inhibition of the beta-catenin pathway. Cancer Lett..

[7] Sadlonova A., Mukherjee S., Bowe D.B., Gault S.R., Dumas N.A., Van Tine B.A., Frolova N., Page G.P., Welch D.R., Novak L. (2007). Human breast fibroblasts inhibit growth of the MCF10AT xenograft model of proliferative breast disease. Am. J. Pathol..

[8] Sadlonova A., Novak Z., Johnson M.R., Bowe D.B., Gault S.R., Page G.P., Thottassery J.V., Welch D.R., Frost A.R. (2005). Breast fibroblasts modulate epithelial cell proliferation in three-dimensional in vitro co-culture. Breast Cancer Res..

[9] Bissell M.J., Hines W.C. (2011). Why don’t we get more cancer? A proposed role of the microenvironment in restraining cancer progression. Nat. Med..

[10] Costa A., Kieffer Y., Scholer-Dahirel A., Pelon F., Bourachot B., Cardon M., Sirven P., Magagna I., Fuhrmann L., Bernard C. (2018). Fibroblast Heterogeneity and Immunosuppressive Environment in Human Breast Cancer. Cancer Cell.

[11] Su S., Chen J., Yao H., Liu J., Yu S., Lao L., Wang M., Luo M., Xing Y., Chen F. (2018). CD10(+)GPR77(+) Cancer-Associated Fibroblasts Promote Cancer Formation and Chemoresistance by Sustaining Cancer Stemness. Cell.

[12] Pelon F., Bourachot B., Kieffer Y., Magagna I., Mermet-Meillon F., Bonnet I., Costa A., Givel A.M., Attieh Y., Barbazan J. (2020). Cancer-associated fibroblast heterogeneity in axillary lymph nodes drives metastases in breast cancer through complementary mechanisms. Nat. Commun..

[13] Orimo A., Gupta P.B., Sgroi D.C., Arenzana-Seisdedos F., Delaunay T., Naeem R., Carey V.J., Richardson A.L., Weinberg R.A. (2005). Stromal fibroblasts present in invasive human breast carcinomas promote tumor growth and angiogenesis through elevated SDF-1/CXCL12 secretion. Cell.

[14] Simonet W.S., Lacey D.L., Dunstan C.R., Kelley M., Chang M.S., Luthy R., Nguyen H.Q., Wooden S., Bennett L., Boone T. (1997). Osteoprotegerin: A novel secreted protein involved in the regulation of bone density. Cell.

[15] Kwon B.S., Wang S., Udagawa N., Haridas V., Lee Z.H., Kim K.K., Oh K.O., Greene J., Li Y., Su J. (1998). TR1, a new member of the tumor necrosis factor receptor superfamily, induces fibroblast proliferation and inhibits osteoclastogenesis and bone resorption. FASEB J..

[16] Infante M., Fabi A., Cognetti F., Gorini S., Caprio M., Fabbri A. (2019). RANKL/RANK/OPG system beyond bone remodeling: Involvement in breast cancer and clinical perspectives. J. Exp. Clin. Cancer Res..

[17] Holen I., Cross S.S., Neville-Webbe H.L., Cross N.A., Balasubramanian S.P., Croucher P.I., Evans C.A., Lippitt J.M., Coleman R.E., Eaton C.L. (2005). Osteoprotegerin (OPG) expression by breast cancer cells in vitro and breast tumours in vivo—A role in tumour cell survival?. Breast Cancer Res. Treat..

[18] Goswami S., Sharma-Walia N. (2016). Osteoprotegerin rich tumor microenvironment: Implications in breast cancer. Oncotarget.

[19] Fisher J.L., Thomas-Mudge R.J., Elliott J., Hards D.K., Sims N.A., Slavin J., Martin T.J., Gillespie M.T. (2006). Osteoprotegerin overexpression by breast cancer cells enhances orthotopic and osseous tumor growth and contrasts with that delivered therapeutically. Cancer Res..

[20] Geerts D., Chopra C., Connelly L. (2020). Osteoprotegerin: Relationship to Breast Cancer Risk and Prognosis. Front. Oncol..

[21] Hawsawi N.M., Ghebeh H., Hendrayani S.F., Tulbah A., Al-Eid M., Al-Tweigeri T., Ajarim D., Alaiya A., Dermime S., Aboussekhra A. (2008). Breast carcinoma-associated fibroblasts and their counterparts display neoplastic-specific changes. Cancer Res..

[22] Al-Mohanna M.A., Al-Khalaf H.H., Al-Yousef N., Aboussekhra A. (2007). The p16INK4a tumor suppressor controls p21WAF1 induction in response to ultraviolet light. Nucleic Acids Res..

[23] Hendrayani S.F., Al-Khalaf H.H., Aboussekhra A. (2014). The Cytokine IL-6 Reactivates Breast Stromal Fibroblasts through Transcription Factor STAT3-dependent Up-regulation of the RNA-binding Protein AUF1. J. Biol. Chem..

[24] Kinugasa Y., Matsui T., Takakura N. (2014). CD44 expressed on cancer-associated fibroblasts is a functional molecule supporting the stemness and drug resistance of malignant cancer cells in the tumor microenvironment. Stem. Cells.

[25] Sun M., Chang Q., Xin M., Wang Q., Li H., Qian J. (2017). Endogenous bone morphogenetic protein 2 plays a role in vascular smooth muscle cell calcification induced by interleukin 6 in vitro. Int. J. Immunopathol. Pharmacol..

[26] Chung S.T., Geerts D., Roseman K., Renaud A., Connelly L. (2017). Osteoprotegerin mediates tumor-promoting effects of Interleukin-1beta in breast cancer cells. Mol. Cancer.

[27] Takai H., Kanematsu M., Yano K., Tsuda E., Higashio K., Ikeda K., Watanabe K., Yamada Y. (1998). Transforming growth factor-beta stimulates the production of osteoprotegerin/osteoclastogenesis inhibitory factor by bone marrow stromal cells. J. Biol. Chem..

[28] Zhang Q., Chen T., Zhang Y., Lyu L., Zhang B., Huang C., Zhou X., Wu Y., Li Z. (2021). MiR-30c-5p regulates adventitial progenitor cells differentiation to vascular smooth muscle cells through targeting OPG. Stem Cell Res. Ther..

[29] Yang X., Lin Y., Shi Y., Li B., Liu W., Yin W., Dang Y., Chu Y., Fan J., He R. (2016). FAP Promotes Immunosuppression by Cancer-Associated Fibroblasts in the Tumor Microenvironment via STAT3-CCL2 Signaling. Cancer Res..

[30] Yu S., Lu Y., Su A., Chen J., Li J., Zhou B., Liu X., Xia Q., Li Y., Li J. (2021). A CD10-OGP Membrane Peptolytic Signaling Axis in Fibroblasts Regulates Lipid Metabolism of Cancer Stem Cells via SCD1. Adv. Sci..

[31] Sun Y., Yang D., Xi L., Chen Y., Fu L., Sun K., Yin J., Li X., Liu S., Qin Y. (2018). Primed atypical ductal hyperplasia-associated fibroblasts promote cell growth and polarity changes of transformed epithelium-like breast cancer MCF-7 cells via miR-200b/c-IKKbeta signaling. Cell Death Dis..

[32] Yang Z., Jin P., Xu S., Zhang T., Yang X., Li X., Wei X., Sun C., Chen G., Ma D. (2018). Dicer reprograms stromal fibroblasts to a pro-inflammatory and tumor-promoting phenotype in ovarian cancer. Cancer Lett..

[33] Smith S.M., Lyu Y.L., Cai L. (2014). NF-kappaB affects proliferation and invasiveness of breast cancer cells by regulating CD44 expression. PLoS ONE.

[34] Wang Y., Liu Y., Huang Z., Chen X., Zhang B. (2022). The roles of osteoprotegerin in cancer, far beyond a bone player. Cell Death Discov..

