# Supplementary material for: Osteoprotegerin (OPG) Upregulation Activates Breast Stromal Fibroblasts and Enhances Their Pro-Carcinogenic Effects through the STAT3/IL-6 Signaling
Source: Cells. 2022 Oct 25;11(21):3369. doi: 10.3390/cells11213369 (PMC9655455; doi:10.3390/cells11213369)
Supplement: Supplementary file 1 [file cells-11-03369-s001.zip › cells-1955781-supplementary.pdf]

**Table S1.** List of primers.

| Oligonucleotides | | |
|---|---|---|
| **Gene symbol** | 5′-forward primer-3′ | 5′-reverse primer-3′ |
| **OPG/*TNFRSF11B*** | *F:* 5′-AAC GGC AAC ACA GCT CAC AAG AAC-3′ | *R:* 5′-TGC TCG AAG GTG AGG TTA GCA TGT-3′ |
| a-**SMA/*ACTA2*** | *F:* 5′-CTA TGC CTC TGG ACG CAC AAC T-3′ | *R:* 5′-CAG ATC CAG ACG CAT GAT GGC A-3′ |
| ***IL-6*** | *F:* 5′-AGA CAG CCA CTC ACC TCT TCA G-3′ | *R:* 5′-TTC TGC CAG TGC CTC TTT GCT G-3′ |
| **SDF-1/*CXCL12*** | *F:* 5′-CTC AAC ACT CCA AAC TGT GCC C-3′ | *R:* 5′-CTC CAG GTA CTC CTG AAT CCA C-3′ |
| ***TGF-***b1 | *F:* 5′-TAC CTG AAC CCG TGT TGC TCT C-3′ | *R:* 5′-GTT GCT GAC GTA TCG CCA GGA A-3′ |
| ***GAPDH*** | *F:* 5′-GAG TCC ACT GGC GTC TTG-3′ | *R:* 5′-GGG GTG CTA AGC AGT TGG T-3 |

**Table S2.** List of antibodies.

| Antibodies | Species | Company | Catalog # |
|---|---|---|---|
| Anti-OPG (E-10) | Mouse | SANTA CRUZ BIOTECHNOLOGY | SC-390518 |
| Anti-CD10 (F-4) | Mouse | SANTA CRUZ BIOTECHNOLOGY | SC-46656 |
| Anti-C5L2 (E-8) | Mouse | SANTA CRUZ BIOTECHNOLOGY | SC-515734 |
| Anti-NF-kB P65 (F-6) | Mouse | SANTA CRUZ BIOTECHNOLOGY | SC-8008 |
| Anti-CD24 (SN3) | Mouse | SANTA CRUZ BIOTECHNOLOGY | SC-19585 |
| Anti-GAPDH (FL-335) | Rabbit | SANTA CRUZ BIOTECHNOLOGY | SC-25778 |
| Anti-pNF-kB P65 (S536) | Rabbit | Cell Signaling | 3031S |
| Anti-pSTAT3 (Y705) (D3A7) XP | Rabbit | Cell Signaling | 9145 |
| Anti-STAT3 (124H6) | Rabbit | Cell Signaling | 9139S |
| Anti-Snail (L70G2) | Mouse | Cell Signaling | 3895S |
| Anti-Nanog (D73G4) XP | Rabbit | Cell Signaling | 4903S |
| Anti-a-SMA | Rabbit | Abcam | ab5694 |
| Anti-FAP-a | Rabbit | Abcam | ab-53066 |
| Anti-IL-6 | Mouse | Abcam | ab-9324 |
| Anti-IL-8 (EP1117Y) | Rabbit | Abcam | ab-52612 |
| Anti-IL-1b | Rabbit | Abcam | ab-2105 |
| Anti-hnRNP/AUF-1 | Rabbit | Abcam | ab50692 |
| Anti-Twist (10E4E6) | Mouse | Abcam | ab-175430 |
| Anti-Vimentin (RV202) | Mouse | Abcam | ab-8978 |
| Anti-CD44 | Rabbit | Sigma | HPA005785 |
| Anti-MMP2 | Mouse | Invitrogen | 436000 |
| Anti-p16 | Mouse | BD Pharmingen | 550834 |
| Anti-ALDH | Mouse | BD Transduction Laboratories | 611195 |
| TGF-b1 (2Ar2) | Mouse | Abcam | ab-64715 |
| SDF-1 | Goat | Abcam | ab-10395 |
